# Nicotinamide mononucleotide as a therapeutic agent to alleviate multi-organ failure in sepsis

**DOI:** 10.1186/s12967-023-04767-3

**Published:** 2023-12-06

**Authors:** Ting Cao, Rui Ni, Weimin Ding, Xiaoyun Ji, Guo-Chang Fan, Zhuxu Zhang, Tianqing Peng

**Affiliations:** 1 Institutes of Biology and Medical Sciences and Institute for Cardiovascular Science, Soochow University, Suzhou, 215123 China; 2 Lawson Health Research Institute, London Health Sciences Centre, London, ON N6A 5W9 Canada; 3 Department of Pathology and Laboratory Medicine, Western University, VRL 6th Floor, A6-140, 800 Commissioners Road, London, ON N6A 4G5 Canada; 4 Department of Pharmacology and Systems Physiology, University of Cincinnati College of Medicine, Cincinnati, OH 45267 USA; 5 Department of Medicine, Western University, London, ON N6A 5W9 Canada

## Abstract

**Background:**

Sepsis-caused multi-organ failure remains the major cause of morbidity and mortality in intensive care units with limited therapeutics. Nicotinamide mononucleotide (NMN), a precursor of nicotinamide adenine dinucleotide (NAD^+^), has been recently reported to be protective in sepsis; however, its therapeutic effects remain to be determined. This study sought to investigate the therapeutic effects of NMN in septic organ failure and its underlying mechanisms.

**Methods:**

Sepsis was induced by feces-injection-in-peritoneum in mice. NMN was given after an hour of sepsis onset. Cultured neutrophils, macrophages and endothelial cells were incubated with various agents.

**Results:**

We demonstrate that administration of NMN elevated NAD^+^ levels and reduced serum lactate levels, oxidative stress, inflammation, and caspase-3 activity in multiple organs of septic mice, which correlated with the attenuation of heart dysfunction, pulmonary microvascular permeability, liver injury, and kidney dysfunction, leading to lower mortality. The therapeutic effects of NMN were associated with lower bacterial burden in blood, and less ROS production in septic mice. NMN improved bacterial phagocytosis and bactericidal activity of macrophages and neutrophils while reducing the lipopolysaccharides-induced inflammatory response of macrophages. In cultured endothelial cells, NMN mitigated mitochondrial dysfunction, inflammation, apoptosis, and barrier dysfunction induced by septic conditions, all of which were offset by SIRT3 inhibition.

**Conclusion:**

NAD^+^ repletion with NMN prevents mitochondrial dysfunction and restrains bacterial dissemination while limiting inflammatory damage through SIRT3 signaling in sepsis. Thus, NMN may represent a therapeutic option for sepsis.

**Supplementary Information:**

The online version contains supplementary material available at 10.1186/s12967-023-04767-3.

## Background

Sepsis-caused multiple-organ failure is the leading cause of death in intensive care unit patients. Despite extensive research, treatment for this fatal condition remains largely supportive without any specifically effective therapies [1].

Nicotinamide adenine dinucleotide (NAD^+^) plays important roles in energy metabolism, cell signalling, gene expression and DNA repair [2]. Previous studies revealed a reduction of NAD^+^ levels in animal models of sepsis [3–5]. Depletion of NAD^+^ may disrupt mitochondrial [6] and lysosomal function [7], and compromise the activities of the NAD^+^ dependent enzymes (e.g. mitochondrial sirtuin (SIRT)3 of the SIRT family) [8], all of which potentially impair host anti-bacterial activity and promote hyper-inflammation and organ failure in sepsis. Thus, NAD^+^ repletion may have therapeutic potential for sepsis. NAD^+^ is generated through three pathways in mammals: the de novo pathway from the L-tryptophan amino acid precursor; the salvage pathway; and the Priess-Handler pathway from precursors such as nicotinamide, niacin, nicotinamide riboside (NR) or nicotinamide mononucleotide (NMN) [2]. Nicotinamide phosphoribosyltransferase (NAMPT), NR kinase 1/2 (NRK1/2), and niacin/NMN adenylyltransferase (NMNAT) are major enzymes within the salvage pathway for NAD^+^ biosynthesis. NAD^+^ precursor supplementation offers a potential mechanism to boost NAD^+^ levels. Although L-tryptophan exhibited some protective effects in sepsis [9], it may compromise immune functions due to its immune-active catabolites [10]. Niacin was reported to attenuate lung inflammation and reduce mortality in endotoxemic rats [4]. Treatment with nicotinamide protected against acute liver injury and increased survival in mouse models of endotoxemia and cecal ligation and puncture-induced sepsis [11]. However, the use of niacin is limited by painful flushing, and nicotinamide may inhibit the SIRT family which is required for essential cellular functions [12, 13]. Nevertheless, these previous studies support the beneficial effects of NAD^+^ repletion in sepsis.

Notably, NR and NMN have the potential to overcome the limitations of these precursors and recent clinical trials supported the safety of both agents in patients [14, 15]. NR and NMN are currently used as health supplements. We found that administration of NR before—but not after—the onset of sepsis prevented organ injury in mice [5], indicating that the use of NR as a therapeutic approach is limited. This may be due to the reduction of NRK1/2 enzymes during sepsis [5] as the salvage of NAD^+^ from NR involves NRK1/2 phosphorylating NR [16]. NMN is one of the intermediates in NAD^+^ biosynthesis and is converted from nicotinamide by NAMPT. It is also found in various types of natural foods, such as vegetables, fruits and meat. There are two forms of NMN—α-NMN and β-NMN—the latter of which is the active form [17]. In contrast to NR, NMN does not require NRK1/2 for its conversion to NAD^+^. A recent study reported that NMN alleviated lipopolysaccharide (LPS)-induced inflammation in macrophages [18]. Most recently, three new papers reported that administration of NMN attenuated LPS-induced lung [19] and kidney injury [20] and ameliorate the dysregulated inflammatory response and reduced mortality in a mouse model of the cecal ligation and puncture-induced sepsis model [21]. These findings strongly suggest that NMN may provide protective effects in sepsis. However, in these studies NMN was given either before LPS treatment or right after CLP surgery. Thus, the therapeutic effects of NMN remain elusive in sepsis.

NAD^+^ is a critical co-factor for SIRT3 activity [8], which prevents the acetylation of key proteins of mPTP (mitochondrial permeability transition pore) [22]. A recent study reported that decreased NAD^+^ impaired SIRT3 function in sepsis [23], leading to mitochondrial dysfunction [24–26]. Accordingly, the inactivation of mPTP provided protective effects in mouse models of sepsis [24, 26, 27]. Since NAD^+^ is an important antioxidant mechanism by directly balancing the redox status and/or indirectly promoting SIRT1/3-mediated antioxidant defences, decreased NAD^+^ may promote mitochondrial dysfunction and organ injury in sepsis through SIRT3 inhibition. However, it remains elusive if NAD^+^ repletion prevents SIRT3 inhibition in alleviating organ dysfunction during sepsis.

This study demonstrates for the first time that the administration of NMN after sepsis onset reduced multi-organ injury and improved survival in septic mice, supporting a therapeutic potential of NMN in sepsis. Mechanistically, the therapeutic effects of NMN may be mediated at least in part by improving SIRT3 signaling and subsequently preventing mitochondrial dysfunction and oxidative stress.

## Materials and methods

### Animals and experimental procedures

This investigation conforms to the Guide for the Care and Use of Laboratory Animals published by the US National Institutes of Health (NIH Publication No. 85–23). All experimental procedures were approved by the Animal Use Subcommittee at Soochow University, China, and Western University, Canada. C57BL/6 were purchased from the Jackson Laboratory.

Sepsis was induced in mice (male at age 2 months) by feces-injection-in-peritoneum (FIP, 3 g feces per kg body weight) as previously described [28]. Mice received the same volume of saline as the sham control. A single dose of NMN (500 mg/kg body weight, Biochempartner, China) or vehicle was intraperitoneally injected into mice after one hour of FIP. Four groups were included: (1) Saline + Vehicle (15 mice); (2) Saline + NMN (15 mice); (3) FIP + Vehicle (15 mice); and (4) FIP + NMN (15 mice). Septic and their relevant sham mice were killed after 6 h of FIP as we previously described [28], and serum, heart, lung, liver, and kidney tissues were harvested for further analyses.

For the 24-h study, a total of 50 mice (male at age 2 months) received an injection of feces (1.8 g/kg, i.p.), and one hour later, they were allocated into two groups: treatment with a single dose of NMN (25 mice, 500 mg/kg body weight, i.p.), or vehicle (25 mice).

For the long-term survival analysis (30 days), a total of 44 mice (male at age 2 months) received an intraperitoneal injection of feces (0.8 g/kg). After one hour of FIP, septic mice were allocated into two groups: treatment with vehicle (22 mice), or NMN (100 mg/kg, 22 mice) every other day for 30 days.

All animals were given sterile saline (1 mL) containing the buprenorphine (4 µg/mL) subcutaneously 30 min prior to feces injection, and then at 8–12 h intervals as appropriate.

### Echocardiography

Animals were lightly anaesthetized with inhaled isoflurane (1%) and imaged on a warm handling platform using a 40 MHz linear array transducer attached to a preclinical ultrasound system (Vevo 2100 and Vevo770, FUJIFILM Visual Sonics, Canada) with a nominal in-plane spatial resolution of 40 μm (axial) × 80 μm (lateral) as we described [29]. Left ventricular (LV) end-systolic inner diameter (LVIDs), LV end-diastolic inner diameter (LVIDd), fractional shortening (FS)%, and ejection fraction (EF)% were analyzed.

### Measurement of pulmonary microvascular permeability

Pulmonary microvascular permeability in mice was assessed using Evans blue dye (EBD, Sigma) as previously described [30]. Briefly, mice were injected with 0.4% Evans blue solution (50 mg/kg) via the caudal vein 30 min before euthanasia. After flashing with 10 mL Phosphate-buffered saline (PBS), the lungs were removed, weighted, and homogenized with PBS. The tissue lysates were mixed with 2 mL of formamide and then incubated at 60 °C for 16 h. The samples were centrifuged at 20,000 g at 4 °C for 5 min, and the supernatant was used to determine the concentration of EBD by spectrophotometry (620/740 nm). The microvascular permeability was assessed by the leak of EBD in lung tissues [EBD (µg)/lung weight (g) × 10/30] (µg EBD/g lung tissue/minute).

### Histological examination

Lung tissues were fixed in 4% paraformaldehyde (Sigma, Canada) at 4 °C for 48 h, and then embedded in paraffin wax, sectioned (5 μm), and processed. A routine hematoxylin and eosin staining was conducted.

### Measurement of AST, ALT, BUN and creatinine

The levels of aspartate transaminase (AST), alanine transaminase (ALT), blood urea nitrogen (BUN), and creatinine in sera were measured using commercial assays following the manufacturer’s instructions (Nanjing Jiancheng Bioengineering Institute, China).

### L-Lactate assay

The L-Lactate level in sera was determined using L-Lactate assay kit (Cayman Chemicals, USA) following the manufacturer’s instructions.

### Measurement of TNF-α, IL-1β, IL-2 and IL-10 proteins

The levels of TNF-α, IL-1β, IL-2 and IL-10 proteins in sera were determined using ELISA kits (Elabscience, China) following the manufacturer’s instructions.

### Measurement of myeloperoxidase (MPO) activity

MPO activity in tissue lysates was determined as previously described [31].

### Cell cultures

Mouse cardiac microvascular endothelial cells (MCECs) were purchased from CELLutions Biosystems (Cedarlane Laboratories, Hornby, ON, Canada) and cultured in DMEM with 10% fetal bovine serum (FBS).

For the isolation of primary peritoneal macrophages, male C57BL/6 mice received an intraperitoneal injection of 2 mL of 3.8% thioglycollate medium. Three days later, 5 mL of Dulbecco’s phosphate-buffered saline (DPBS) was injected into the mouse peritoneum and then the peritoneal lavage fluid was harvested. After a brief centrifugation, the peritoneal macrophage pellet was re-suspended and cultured in DMEM with 10% FBS for the study.

Bone marrow-derived neutrophils were isolated from male C57BL/6 mice and cultured according to a previous study [32].

### Preparation of LPS-conditioned medium

Mouse macrophage cell line (RAW264.7) was obtained from ATCC (USA) and cultured in DMEM containing 10% FBS (Thermo Fisher Scientific Inc.). After incubation with LPS (100 ng/mL in culture medium) for 24 h, the culture media (LPS-conditioned medium, LCM) were collected and used to simulate septic conditions for the following in vitro studies. PBS-conditioned culture medium (PCM) from RAW264.7 cells served as a control.

### Cell counting kit-8 (CCK-8)

Cell viability was assessed using the CCK-8 assay kit (Nanjing Jiancheng Bioengineering Institute, China) following the manufacturer’s instructions.

### Endothelial cell barrier function

Endothelial cell barrier function was determined in MCECs as described previously [33]. In brief, monolayer MCECs in 24-well plate transwell inserts (pore diameter size 0.4 µm, Corning Costar, USA) were incubated with a culture medium containing NMN or a vehicle for 12 h, and then exposed to LCM medium or PCM for 24 h. Culture medium in the transwell inserts (the upper chambers) was replaced with 50 µL of 0.66% Evans blue solution (dissolved in 4% bovine serum albumin, BSA) while the bottom chambers were balanced with 500 µL of 4% BSA. One hour later, the solution collected from the bottom chambers was examined by spectrophotometric analysis (absorbance at 650 nm).

### Assessment of the mitochondrial permeability transition pore (mPTP) opening

The mPTP opening was determined using Calcein AM (Thermo Fisher Scientific, USA) following the manufacturer’s instructions as previously described [34]. The nuclei were stained using Hoechst 33,342.

### Determination of caspase-3 activity

Caspase-3 activity in endothelial cells and tissue lysates was determined using a caspase-3 fluorescent assay kit (BIOMOL Research Laboratories) following the manufacturer’s instructions.

### Reactive oxygen species (ROS) production

The production of hydrogen peroxide in organ tissues was measured using the Amplex Red Hydrogen Peroxide/Peroxidase assay kit (Thermo Fisher Scientific, USA) following the manufacturer’s instructions.

Mitochondrial superoxide production in live endothelial cells was determined using Mito-SOX™ Mitochondrial Superoxide Indicator (Thermo Fisher Scientific, USA) according to the manufacturer’s instructions.

### Assessment of malondialdehyde (MDA) production and carbonyl protein content

The levels of MDA and carbonyl protein content in tissue lysates were determined using a TBARS assay kit and Protein Carbonyl Colorimetric Assay Kit (Cayman Chemical Company, USA), respectively, following the manufacturer’s instructions.

### ***Determination of NAD***^+^***levels***

NAD^+^ levels in cell and tissue lysates were measured using NAD/NADH Microplate Assay Kits (Cohesion Biosciences, UK, and Beyotime, China) following the manufacturer’s instructions.

### Determination of ATP production

The levels of ATP production in cell and tissue lysates were measured using the ATP Bioluminescence Assay Kit (Beyotime, China), according to the manufacturer’s instructions.

### Real-time RT-PCR

Total RNA from cell and tissue lysates were extracted using TRIzol reagent (Sigma Aldrich, USA) following the manufacturer’s instructions. Real-time reverse transcription PCR (RT-PCR) was performed to analyze the mRNA levels of NRK1, NRK2, Slc12a8, TNF-α, IL-1β, iNOS, VCAM1 and GAPDH. Primers used for RT-PCR are listed in Additional file 1: Table S1. GAPDH served as a loading control. The mRNA levels of individual genes were expressed as the ratio relative to GAPDH.

### Bacterial burden assay

Whole blood and peritoneal lavage fluid were harvested from septic mice. A bacterial burden assay was performed as described previously [35].

### In vivo* phagocytosis assay*

The bacterial uptake by neutrophils in vivo was determined as previously described [36]. Briefly, 0.2 mg of pHrodo^™^ Green *E. coli* BioParticles (dissolved in 200 µL PBS, Invitrogen/Thermo Fisher, MA, USA) was injected into the mouse peritoneal cavity at 4 and 22 h after FIP, respectively. After 2 h of *E. coli* BioParticle injection, the animals were euthanized and their peritoneal lavages were collected. To block Fc-receptors, the cells from peritoneal lavage fluids were incubated with an anti-FcγR-blocking antibody (clone 2.4G2) on ice for 20 min. Then the cells were stained with APC/Cy7 anti-mouse CD45 antibody (clone 30-F11, leukocyte marker), PE anti-mouse CD11b antibody (clone M1/70, myeloid cell marker) and PE/Cy7 anti-mouse Ly-6G antibody (clone 1A8, neutrophil marker) on ice for 30 min. All antibodies were purchased from BioLegend (USA). The phagocytosis of pHrodo Green *E. coli* Bioparticles by neutrophils (CD45^+^CD11b^+^Ly6G^+^) was determined by Flow cytometry analysis (FACSCanto^™^ II, BD biosciences, USA). A total of 20,000 events were measured per mouse and analyzed with FlowJo 7 software (Tree Star).

### In vitro* phagocytosis assay with fluorescence-labelled bacteria*

To analyze the phagocytic capacity of macrophages, primary peritoneal macrophages from mice were seeded in 96 well plates (5 × 10^4 cells/well). Four hours later, macrophages were incubated with NMN or vehicle for an hour, followed by the addition of pHrodo^™^ Green *E. coli* BioParticles (Invitrogen/Thermo Fisher, MA, USA). The fluorescence intensity was measured using a Hybrid Multi-mode microplate reader (BioTek; excitation/emission: 485/535 nm) at different time points (0.5, 1 and 2 h) after addition of pHrodo^™^ Green *E. coli* BioParticles.

Neutrophils isolated form bone marrow were treated with NMN or vehicle for an hour at 37 ℃ with end-over-end-rotation (10 rpm) and then incubated with pHrodo™ Green *E. coli* BioParticles under the same conditions for different times (0.5, 1 and 2 h). Thereafter, the cells were collected and fixed with 2% paraformaldehyde for 15 min on ice. The uptake of pHrodo™ Green *E. coli* Bioparticles by neutrophils was determined by Flow cytometry analysis (FACSCantoTM II, BD biosciences, USA). A total of 20,000 events was measured per mouse and analyzed with FlowJo 7 software (Tree Star).

### In vitro* bacterial phagocytosis and killing assay*

A classical colony-forming unit (CFU) assay was conducted as described previously with minor modifications [37]. Briefly, *E. coli* ATCC 25922 strain was grown in Luria–Bertani broth at 37 °C. After being quantified, pelleted, and washed with PBS, the bacteria were ready for the following studies.

Primary peritoneal macrophages (4 × 10^5 cells) were seeded in 12-well plates. Four hours later, macrophages were treated with NMN or a vehicle and then challenged with *E. coli* ATCC 25922 strain (multiplicity of infection: 20) at 37 °C for one hour. Culture medium was then replaced by fresh one containing 100 μg/mL of gentamicin and the cells were incubated for 30 min to kill extracellular bacteria. After washing, macrophages were either lysed with 0.05% saponin to determine the uptake of bacteria by macrophages or incubated with normal culture medium for 5 more hours to assess their bactericidal activities. For the phagocytosis, the macrophage lysates were diluted and plated on Luria–Bertani plates to determine the colony forming units (CFUs). To assess the bactericidal activity, the number of living bacteria that remained after the intracellular killing was determined by lysing cells and then plating the cell lysates on Luria–Bertani plates as previously described [35]. The killing percentage was calculated using the formula {[(CFU counts at one hour)–(CUF count at 6 h)]/(CFU count at 1 h)} × 100%.

For neutrophil bacterial phagocytosis and killing assay, a total of 1 × 10^5 freshly isolated neutrophils were incubated with NMN or vehicle at 37 ℃ with end-over-end-rotation (10 rpm) for an hour. After adding freshly opsonized *E. coli* ATCC 25922 strain (multiplicity of infection: 20), the neutrophils were continually cultured at the same conditions. Five minutes later, the cells were placed on ice and then centrifuged (100 g) at 4 ℃ for 5 min. After washing the pellets twice with 1 ml of ice-cold PBS for each, half of them were used for the bacterial phagocytosis assay and the remaining half of neutrophils for the bacterial killing assay. To determine the uptake of bacteria, the pellets were re-suspended in 0.9 ml of 0.05% saponin and passed through a 26-gauge syringe needle for five times. Subsequently, 2 μl DNase mix (50 U/μl, Beyotime) containing 100 μl reaction buffer (100 mM Tris–HCl pH 7.4, 1 mM CaCl_2_, 25 mM MgCl_2_) was added to the cell lysates. After incubation at 37 ℃ for 10 min, the cell lysates were plated on the LB plates with appropriate dilutions to determine the CFUs. To assess the bactericidal activity, neutrophils were incubated at 37 ℃ for 2 h. The remaining viable bacteria after intracellular killing were determined as described in above. The killing percentage was calculated using the formula {[(CFU count at 5 min)–(CUF count at 2 h)]/(CFU count at 5 min)} × 100%.

### Statistical analysis

All data are presented as mean values ± SD. One-way analysis of variance followed by a Newman–Keuls test was performed for multi-group comparisons, as appropriate. Student t-test was used for 2-group comparison. Survival curves were created by the method of Kaplan and Meier and compared by the log-rank test. A value of *P* < 0.05 was considered statistically significant.

## Results

### ***NMN boosts NAD***^+^***levels in sepsis***

**Fig. 1:**
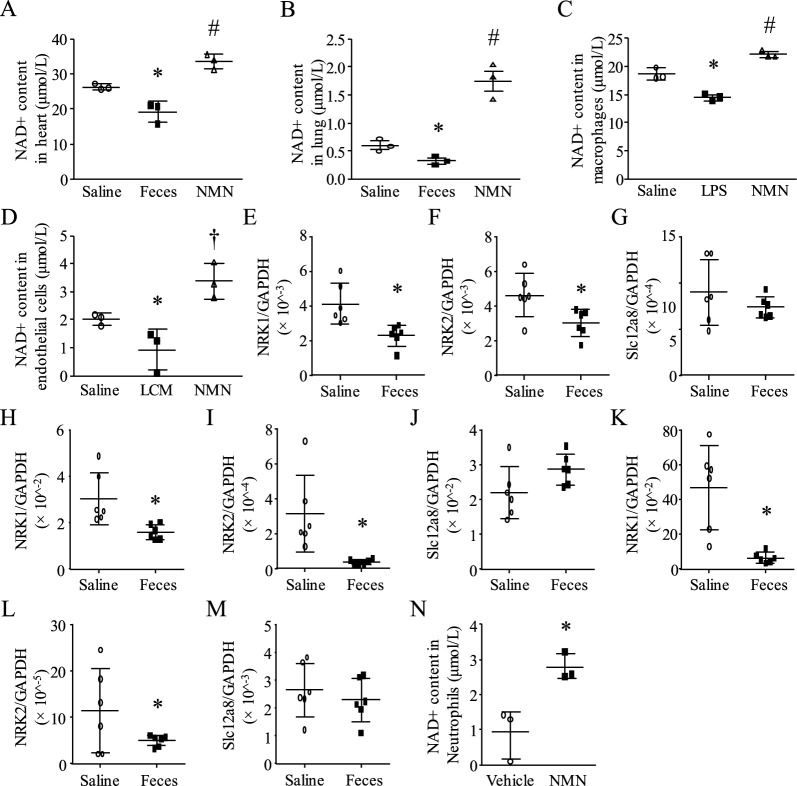
NAD^+^ content, NRK1/2 and Slc12a8 expression. A and B Mice received saline, FIP or NMN (500 mg/kg, i.p.). Six hours later, NAD^+^ levels in heart (**A**) and lung tissues (**B**) were determined. **C** Macrophages were treated with NMN (500 µM), LPS (100 ng/mL) or saline. NAD^+^ levels were analyzed. **D** Endothelial cells were treated with LPS-conditioned medium (LCM), PBS-conditioned medium (PCM) or NMN. Twenty-four hours later, NAD^+^ levels in endothelial cells were measured. **E**–**M** Mice were challenged with FIP or saline. Six hours later, heart, lung and liver tissues were collected. The mRNA levels of NRK1/2 and Slc12a8 were quantified by real-time PCR in heart (**E**–**G)**, lung (**H**–**J**) and liver tissues (**K**–**M**). (**N**) Neutrophils were incubated with NMN for 24 h and NAD^+^ were then determined in neutrophils. Data are mean ± SD, n = 3–6 in each group. *P < 0.05 vs saline or vehicle and #*P* < 0.05 vs saline, feces or LCM (One-way analysis of variance followed by Newman–Keuls test)

We and others have reported that sepsis resulted in a reduction of NAD^+^ levels in the liver and lung tissues of animal models of sepsis [3–5]. Consistently, we showed that the levels of NAD^+^ were much lower in heart and lung tissues in a mouse model of FIP-induced sepsis compared with sham animals (Fig. 1A and 1B). Furthermore, LPS treatment resulted in lower NAD^+^ levels in macrophages, and endothelial cells incubated with LCM (1:1 dilution in normal culture medium) had lower NAD^+^ levels compared with PCM (1:1 dilution in normal culture medium) (Fig. 1C and 1D). These results provide both in vivo and in vitro evidence confirming that septic conditions reduce NAD^+^ levels.

Both NR and NMN have been widely used to boost NAD^+^ levels [38]. NR requires NRK1/2 for its conversion to NAD^+^ within cells, while NMN can enter the cells through its receptor Slc12a8 [39]. As shown in Fig. 1E-1M, sepsis resulted in significantly lower levels of NRK1/2 mRNA, but not S1c12a8, in the heart, lung, and liver within 6 h after FIP. These results provided a rationale for the use of NMN, but not NR, as a therapy for sepsis. Administration of NMN (500 mg/kg) increased NAD^+^ levels in heart and lung tissues (Fig. 1A and 1B). This dosage of NMN has been used in animal models by others [40]. Incubation with NMN (500 µM) also elevated NAD^+^ levels in macrophages, endothelial cells, and neutrophils under normal and/or septic conditions (Fig. 1C, 1D and 1N). These results demonstrate that NMN effectively boosts NAD^+^ levels in sepsis.

### Therapeutic administration of NMN mitigates multi-organ injury and improves survival in mouse models of sepsis

Six hours after FIP, the levels of L-Lactate were significantly elevated in sera, which was attenuated by NMN (Additional file 1: Figure S1A). NMN treatment significantly reduced the protein levels of proinflammatory cytokines TNF-α and IL-1β (Additional file 1: Figure S1B and C) but not anti-inflammatory cytokines IL-2 and IL-10 in plasma of septic mice at 6 h after FIP (Additional file 1: Figure S1D and E).

**Fig. 2:**
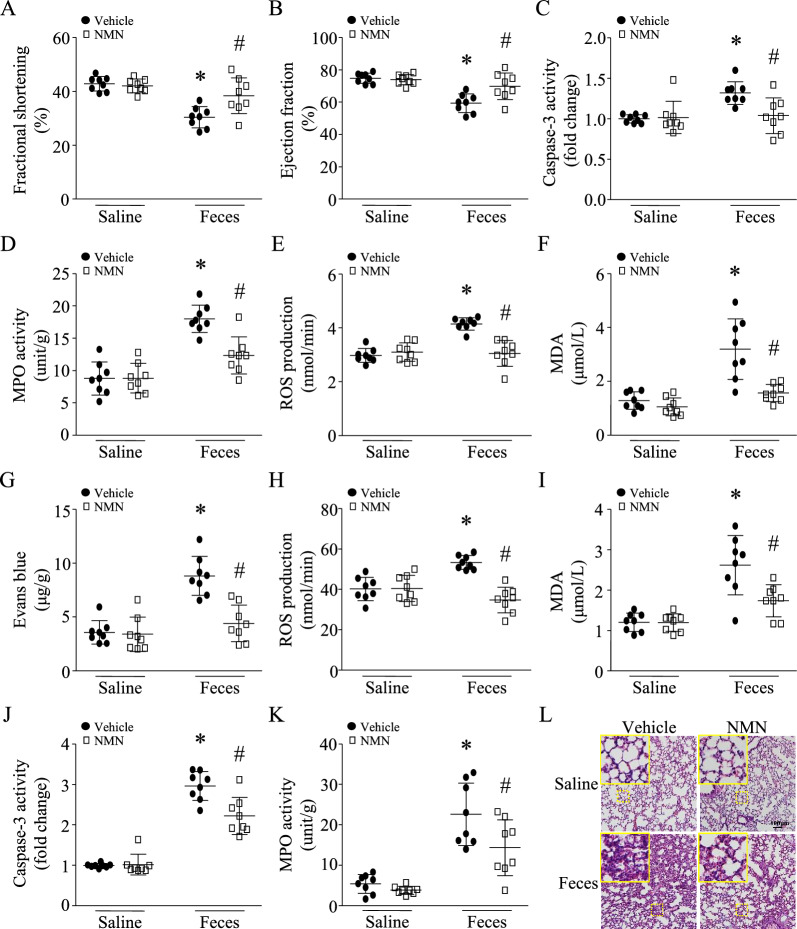
Therapeutic effects of NMN on heart and lung injury in septic mice. After an hour of FIP (3 g feces/kg body weight), mice received a single dose of NMN (500 mg/kg, i.p.) or vehicle. Six hours after FIP, myocardial function (**A** and **B**), caspase-3 activity (**C**), MPO activity (**D**), ROS production (**E**) and MDA level (**F**) were determined in the heart. **G** Pulmonary vascular permeability was assessed by Evans blue assay. ROS production (**H**), MDA level (**I**), caspase-3 activity (**J**) and MPO activity (**K**) were determined in lung tissues. **L** Lung tissues were fixed, embedded and sectioned. H&E staining was performed. A representative microphotograph for H&E staining from 6 lung samples is presented. Data are mean ± SD, n = 8 mice in each group. *P < 0.05 vs saline + vehicle and #*P* < 0.05 vs feces + vehicle (One-way ANOVA followed by Newman–Keuls test)

Echocardiographic analysis revealed that administration of NMN relatively preserved fractional shortening (FS)% and ejection fraction (EF)% in septic mice (Fig. 2A and 2B). NMN also reduced apoptosis, indicated by caspase-3 activity (Fig. 2C), infiltration of inflammatory cells (i.e. neutrophils) as assessed by myeloperoxidase (MPO) activity (Fig. 2D), and oxidative stress as determined by reactive oxygen species (ROS) and malondialdehyde (MDA) production in septic mouse hearts (Fig. 2E and 2F).

Sepsis increased the pulmonary vascular permeability, as determined by the Evans blue assay (Fig. 2G), resulted in more ROS and MDA production (Fig. 2H, I), promoted caspase-3 activity (Fig. 2J), and increased MPO activity in lung tissues (Fig. 2K). These effects of sepsis were attenuated by NMN in septic mice. Histological analysis revealed lung injury in septic mice manifested by highly inflammatory cell infiltration, alveolar wall edema and thickness in the lung (Fig. 2L). These pathological changes were attenuated by NMN in septic mice.

**Fig. 3:**
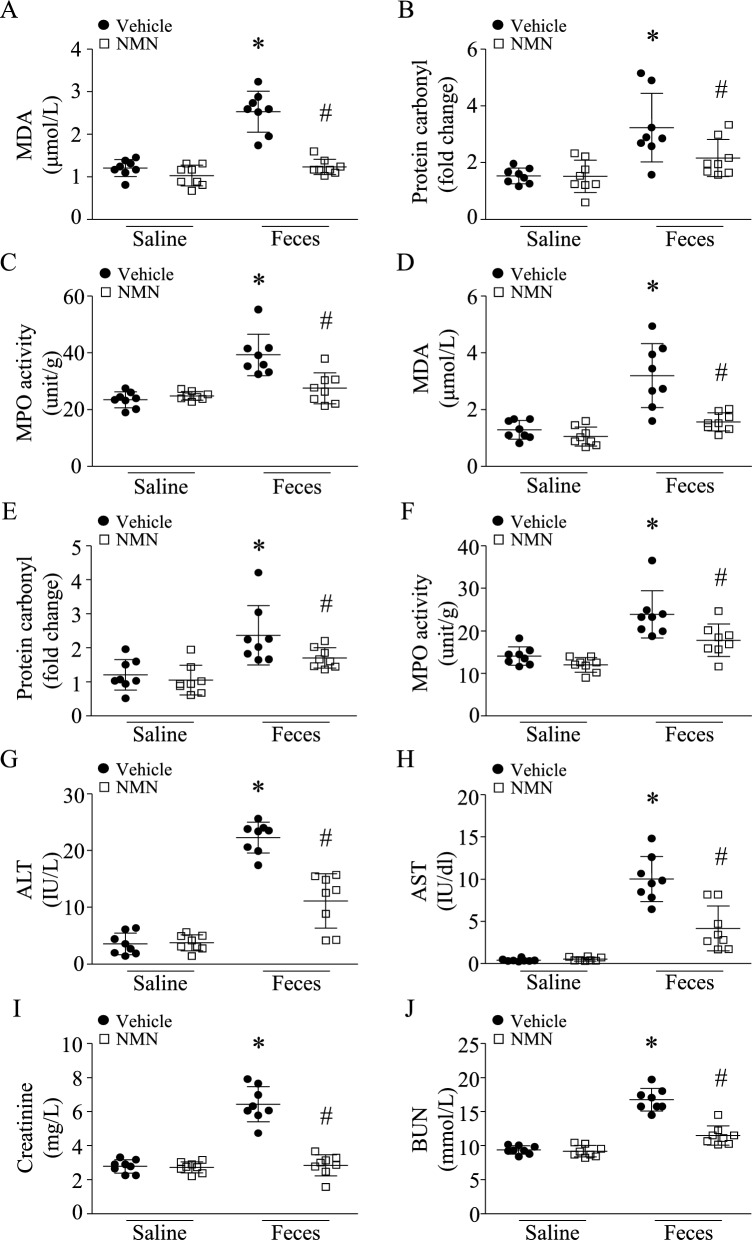
Therapeutic effects of NMN on liver and kidney injury in septic mice. After an hour of FIP (3 g feces/kg body weight), mice were intraperitoneally administrated with NMN. Six hours after FIP, blood and liver and kidney tissues were harvested. **A**–**C** MDA level, protein carbonyl and MPO activity were determined in liver tissues. **D**-**F** MDA level, protein carbonyl and MPO activity were determined in kidney tissues. ALT (**G**), AST (**H**), creatinine (**I**) and BUN (**J**) in sera were measured. Data are mean ± SD, n = 8 mice in each group. *P < 0.05 vs saline + vehicle and #*P* < 0.05 vs feces + vehicle (One-way ANOVA followed by Newman–Keuls test)

Administration of NMN prevented oxidative stress, as assessed by MDA and protein carbonyl production, and reduced MPO activity in the liver (Fig. 3A-3C) and kidney of septic mice (Fig. 3D-3F). NMN treatment also decreased serum levels of ALT, AST, creatinine, and BUN in septic mice (Fig. 3G-3J), indicating the attenuation of liver and kidney damage, respectively.

**Fig. 4:**
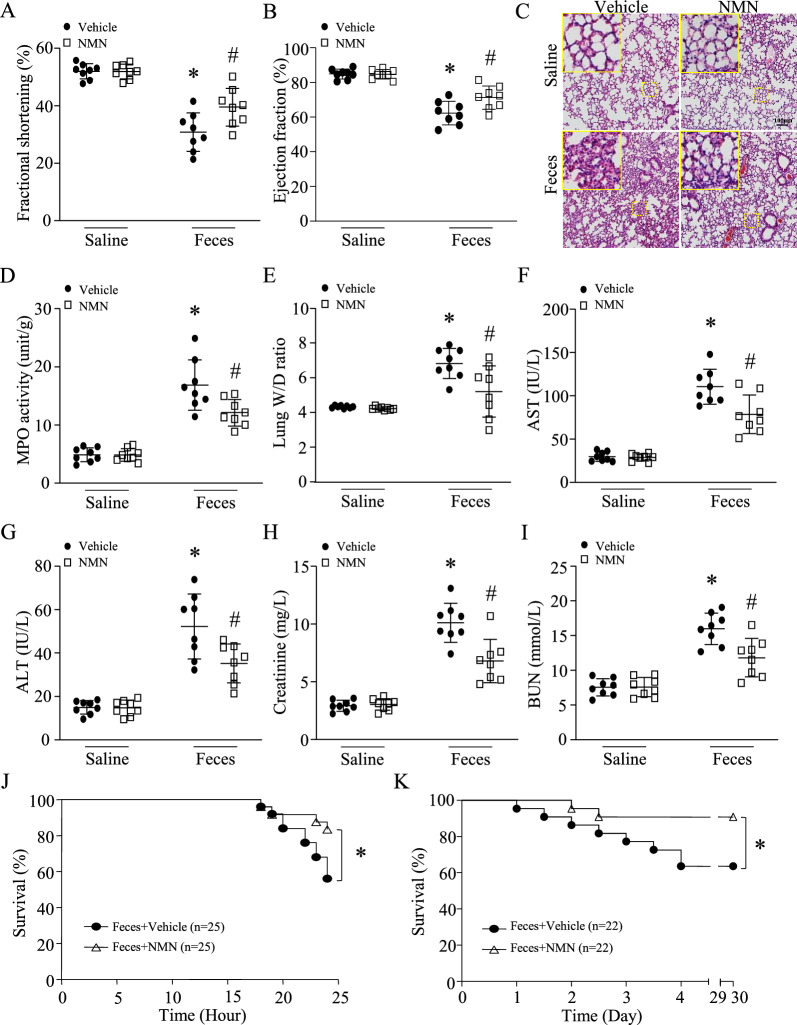
Therapeutic effects of NMN in septic mice. After an hour of FIP (1.8 g feces/kg body weight), mice received a single dose of NMN (500 mg/kg, i.p.) or vehicle. Twenty-four hours after FIP, myocardial function was assessed by echocardiography (**A** and **B**). (**C**) Lung tissues were fixed, embedded and sectioned. H&E staining were performed. A representative microphotograph for H&E staining from 8 lung samples is presented. **D** MPO activity in lung tissues. **E** The ratio of wet lung/dry lung. **F** and **G** AST and ALT in serum. (**H** and **I**) Creatinine and BUN in serum. Data are mean ± SD, n = 8 mice in each group. *P < 0.05 vs saline + vehicle and #*P* < 0.05 vs feces + vehicle (One-way ANOVA followed by Newman–Keuls test). **J** A total of 50 mice (male at age 2 months, 25 mice in each group) were monitored for survival for 24 h after FIP (1.8 g feces /kg body weight). *P < 0.05 (the log-rank test). **K** A total of 44 mice (male at age 2 months) received an intraperitoneal injection of feces (0.8 g/kg). After one hour of FIP, septic mice were allocated into two groups: treatment with vehicle (22 mice) or NMN (100 mg/kg, 22 mice) every other day for 30 days. The survival was monitored. *P < 0.05 (the log-rank test)

To examine the therapeutic effects of NMN at 24 h after FIP, we induced sepsis in mice using a lower dose of feces (1.8 g/kg, i.p.) because our preliminary study found that injection of 3 g feces/kg body weight into mice resulted in 100% mortality within 24 h. A single dose of NMN (500 mg/kg, i.p.) was given after one hour of FIP. Twenty-four hours after FIP, NMN treatment reduced the levels of L-Lactate levels in serum (Additional file 1: Figure S1F) and provided a similar protection against heart (Fig. 4A, B), lung (Fig. 4C, D, E), liver (Fig. 4F, G) and kidney injury in a mouse model of FIP-induced sepsis (Fig. 4H, I). Consequently, NMN treatment reduced mortality in septic mice from 44 to 16% (Fig. 4J, n = 25 mice in each group, *P* < 0.05). The body surface temperature decreased after FIP; however, NMN treatment slightly increased the body temperature in septic mice (Additional file 1: Figure S2).

To determine the effect of NMN on long-term mortality, septic mice received the first dose of NMN (100 mg/kg) after one hour of FIP (0.8 g feces /kg body weight, i.p.) and then NMN injection was followed every other day for a total of 30 days. To provide evidence of successful modeling as a low dosage of feces was used, we analyzed organ dysfunction/injury and systemic inflammation in mice 24 h after FIP. All septic mice displayed similar patterns of organ injury/dysfunction (myocardial dysfunction, lung injury, and liver and kidney injury/dysfunction) with different degrees and an increase in inflammatory cytokines in serum (Additional file 1: Table S2 and Additional file 1: Figure S3). As shown in Fig. 4K, the 30-day mortality was reduced from 36 to 9% by NMN treatment (n = 22 mice in each group, *P* < 0.05). The doses of NMN from 100 to 500 mg/kg were based on previous reports [40]. Importantly, the dose of NMN (100 mg/kg) in mice is equivalent to about 500 mg for human (60–70 kg body weight) [41], which was found to be safe in a recent clinical trial [15]. Thus, our results indicate that treatment with NMN improves survival in sepsis.

### NMN reduces bacterial burden and enhances phagocytic and bactericidal activities of neutrophils and macrophages

**Fig. 5:**
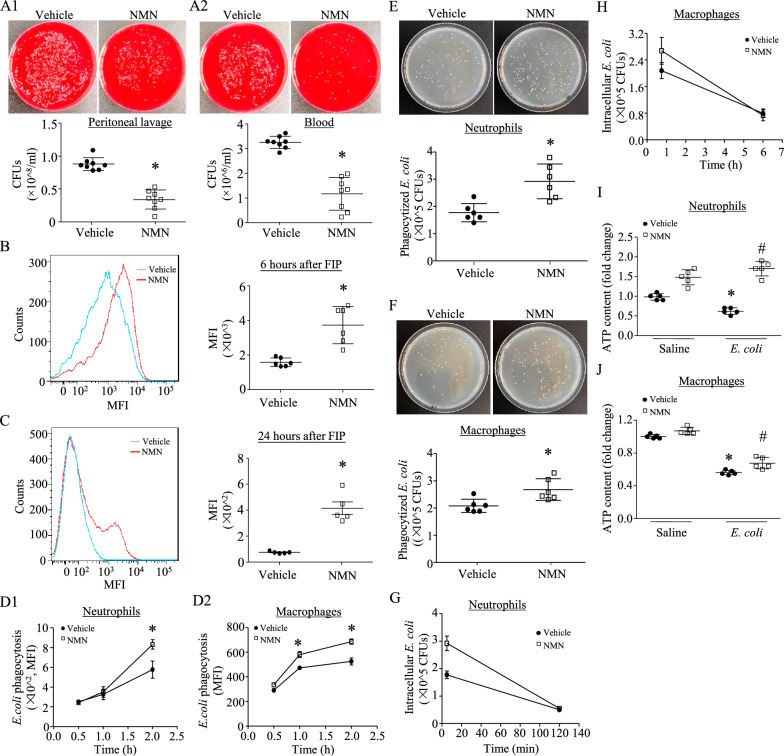
Effects of NMN on phagocytic and bactericidal activity and ATP production. **A**, **B**, **C** A single dose of NMN (500 mg/kg, i.p.) was administrated into mice after an hour of FIP. Twenty-four hours later, the peritoneal lavage fluids and blood were assayed for bacterial loads. **A** Upper panels: representative pictures of bacterial colonies from peritoneal lavage (A1) and blood on plates (A2); Lower panel: colony forming units (CFUs) in each group. Data are mean ± SD, n = 8 mice in each group. *P < 0.05 vs vehicle. **B** and **C** In vivo neutrophil phagocytosis in peritoneum was analyzed at 6 h (**B**) and 24 h after FIP, respectively. Left panels: representative flow cytometry analysis of neutrophil uptake of pHrodo Green *E. coli* Bioparticles from 5–6 mice in each group; Right panels: quantification of fluorescence intensity. Data are mean ± SD, n = 5–6 mice in each group. *P < 0.05 vs vehicle. **D**, **E**, **F**, **G**, **H** In vitro phagocytosis and bacterial killing.(**D** Phagocytosis was determined in neutrophils (D1) and macrophages (D2) at different time points (0.5, 1 and 2 h) by analyzing the uptake of pHrodo Green *E. coli* Bioparticles. **E** and **F** Neutrophils and macrophages were pretreated with NMN (500 µM) and then incubated with living *E. coli* bacteria for 5 min and one hour, respectively. Upper panels: representative pictures of engulfed *E. coli* on the tryptic agar plates; Lower panel: CFUs of *E. coli* in each group (E for neutrophils and F for macrophages). **G** and **H** *E. coli* killing assay. Neutrophils and macrophages were pretreated with NMN (500 µM) and then incubated with living *E. coli* bacteria for 5 min or one hour, respectively. After washing neutrophils or incubating macrophages with gentamicin for 30 min to remove extracellular *E. coli* bacteria, the intracellular living bacteria were determined as described above. After that, the neutrophils and macrophages were maintained in normal cultured medium at 37℃ for additional 2 and 5 h, respectively. The intracellular living bacteria were then analyzed (**G** for neutrophils and H for macrophages). MFI, median fluorescence intensity. **I**, **J** ATP production in neutrophils and macrophages. Cells were pretreated with NMN and then challenged with *E. coli*. ATP production was determined in neutrophils (**I**) and macrophages (**J**). Data are mean ± SD from 5 to 8 independent experiments. *P < 0.05 vs vehicle or saline + vehicle and #* P* < 0.05 vs *E. coli* + vehicle (One-way ANOVA followed by Newman–Keuls test)

After 24 h of FIP, administration of NMN significantly reduced the bacterial loads in peritoneal lavage and blood of septic mice (Fig. 5A). The reduction of bacterial burden was associated with an increase in bacterial phagocytosis of peritoneal neutrophils at 6 and 24 h after FIP, respectively, as determined by an in vivo bacterial uptake assay using pHrodo^™^ Green *E. coli* BioParticles (Fig. 5B and 5C, Additional file 1: Figure S4). To further address the inhibitory effects of NMN on bacterial dissemination, we conducted bacterial phagocytosis and intracellular killing assays using primary macrophages and neutrophils isolated from mouse peritoneum and bone marrow, respectively, as both cells play critical roles in antibacterial defences in sepsis. First, we examined the effect of NMN on cell viability. NMN (500 µM) did not affect the cell viability in neutrophils and macrophages under normal conditions or in the presence of *E*. *coli* bacteria (Additional file 1: Figure S5A and S5B). We then used the pHrodo™ Green *E. coli* BioParticles to assess bacterial phagocytosis of neutrophils and macrophages. As shown in Fig. 5D1, D2, treatments with NMN resulted in a higher uptake of *E*. *coli* BioParticles in neutrophils and macrophages, suggesting that NMN increases bacterial phagocytosis. To further address this, we analyzed the phagocytic and bactericidal activities of neutrophils and macrophages using living *E*. *coli* bacteria. We showed that the NMN treatment increased bacterial uptake in neutrophils and macrophages by about 65% and 28.8%, respectively, when compared to vehicle-treated cells (Fig. 5E and 5F, Additional file 1: Table S3 and S4). Notably, NMN treatment resulted in higher bacterial killing activity in both neutrophils (from 72.14% to 81.13%) and macrophages (from 61.40% to 72.46%) compared to that of vehicle-treated cells (Fig. 5G and H, Additional file 1: Table S3 and S4). The increases in bacterial phagocytosis and killing activity were associated with more ATP production in NMN-treated neutrophils and macrophages (Fig. 5I and 5J). Thus, treatment of neutrophils and macrophages with NMN could improve their phagocytosis and bactericidal activities.

Since macrophages also significantly contribute to systemic inflammatory response during sepsis, we examined the effects of NMN on LPS-induced inflammatory cytokine expression in macrophages. Primary peritoneal macrophages were incubated with LPS (100 ng/mL) or saline in the presence of NMN (500 µM) or a vehicle for 24 h. LPS treatment resulted in significantly higher levels of TNF-α and IL-1β mRNA in macrophages, which was attenuated by NMN (Additional file 1: Figure S6A and 6B). This result suggests that NMN may limit the inflammatory response in macrophages under septic conditions.

### NMN reduces LPS-conditioned medium-induced endothelial cell dysfunction and death

**Fig. 6:**
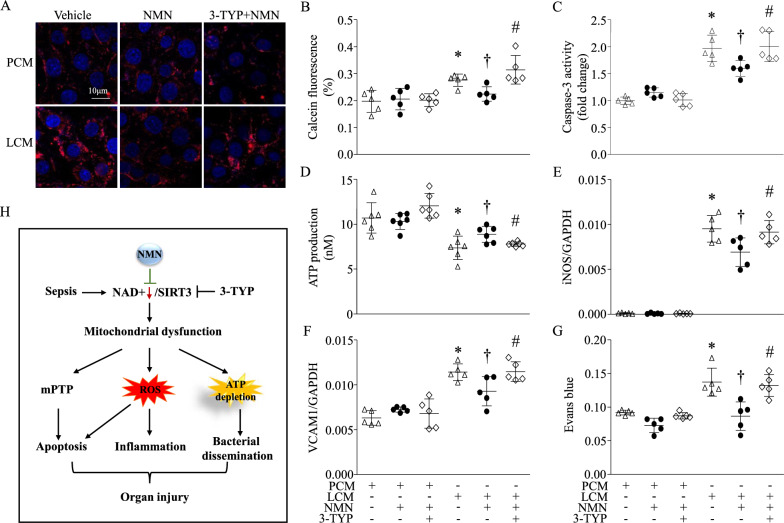
Effects of NMN on endothelial cell dysfunction and death. Conditioned medium collected from RAW264.7 cells stimulated with LPS (LCM) for 24 h was used to simulate septic conditions. Medium from PBS-stimulated RAW264.7 cells served as a control medium (PCM). Mouse cardiac microvascular endothelial cells (MCECs) were exposed to LCM or PCM in the presence of NMN, 3-TYP or a vehicle for 24 h. **A** Mitochondrial ROS generation was measured in living MCECs by Mito-SOX staining. A representative microphotograph for Mito-SOX staining (red colour) and nuclear staining with Hoechst 33,342 (blue colour) from 3 independent experiments is presented. **B** Mitochondrial permeability transition pore (mPTP) opening was assessed using Calcein fluorescence dye. Caspase-3 activity (**C**) and ATP production (**D**) were determined in cell lysates. The mRNA levels of iNOS (**E**) and VCAM1 (**F**) relative to GAPDH were analyzed by real-time PCR. **G** Monolayer cell permeability was assayed by the leakage of Evans blue dye. Data are mean ± SD from 3–6 different cell cultures. *P < 0.05 vs PCM, †*P* < 0.05 vs LCM, and #*P* < 0.05 vs LCM + NMN (One-way ANOVA followed by Newman–Keuls test). **H** Schematic mechanisms for the protective effects of NMN on septic organ injury

Since endothelial dysfunction has been critical in septic organ dysfunction [42], we determined the protective effects of NMN in cardiac microvascular endothelial cells. MCECs were incubated with LCM or PCM (1:1 dilution in normal culture medium) in the presence of NMN (500 µM) or vehicle for 24 h. This dosage of NMN was chosen based on the dose-dependent cytotoxic effect of NMN in MCECs (Additional file 1: Figure S7). LCM-induced MCECs had higher levels of mitochondrial ROS production (Fig. 6A), mPTP opening (Fig. 6B), and caspase-3 activity (Fig. 6C), and lower levels of ATP production (Fig. 6D) compared with PCM, all of which were prevented by NMN. LCM resulted in inflammatory responses indicated as iNOS and VCAM1 mRNA expression. NMN attenuated higher levels of iNOS and VCAM1 mRNA in LCM-induced MCECs (Fig. 6E and 6F). NMN prevented endothelial cell barrier dysfunction induced by LCM as demonstrated by increased monolayer cell permeability (Fig. 6G). These results indicate that NMN protects endothelial cell function and prevents cell death under septic conditions. As NAD^+^ is required for SIRT3 activation in mitochondria, we found that with a selective inhibitor of SIRT3, 3-TYP (100 µM), NMN was unable to prevent LCM-induced mitochondrial ROS production, deletion of ATP production, mPTP opening, apoptosis, inflammation and barrier dysfunction in MCECs (Fig. 6A-6G), supporting the involvement of SIRT3 signalling.

## Discussion

Although NMN has been recently reported to be protective in preclinical animal models of endotoxemia and CLP-induced sepsis [19–21], the present study demonstrates for the first time that therapeutic administration of NMN through NAD^+^ repletion reduces major organ dysfunction and improves survival in septic mice. The therapeutic effects of NMN are associated with an enhancement of the bactericidal activity of macrophages and neutrophils, suppression of hyperinflammatory response, and prevention of inflammatory damage to organs during sepsis. Thus, this study provides the first experimental evidence to support that NMN may be an effective therapy to protect organs in sepsis and minimize mortality.

### ***NAD***^+^***repletion with NMN enhances bactericidal activity of neutrophils and macrophages while limiting hyperinflammation in sepsis***

The phagocytosis and internal killing of pathogens by phagocytic cells—particularly neutrophils and macrophages—are critical in limiting the dissemination of pathogens during sepsis. It has been reported that patients with defects in phagocytic function typically experience early dissemination of pathogens, which is closely associated with severe sepsis and higher mortality [43]. Reduced phagocytic activity during the first 24 h after admission positively correlated with mortality in septic patients [44]. As professional phagocytic cells, neutrophils and macrophages play important roles in host defence against pathogens because of their ability to internalize and kill bacteria. Accordingly, the dysfunction of macrophages and neutrophils contribute to insufficient antibacterial defences and hyperinflammation in sepsis [45–48]. We recently discovered that Sectm1a enhances bacterial phagocytosis and bactericidal activity of macrophages, and reduces inflammation, thereby improving survival in sepsis [35, 49, 50]. Thus, maintaining or enhancing the bactericidal activity of neutrophils and macrophages may be an alternative treatment strategy for sepsis. In line with this, the present study showed that administration of NMN significantly reduced the bacterial loads in peritoneal lavage and blood of FIP mice, suggesting an inhibitory effect of NMN on bacterial dissemination in sepsis. This may result from an enhancement of phagocytic and bactericidal activities of neutrophils and macrophages by NMN, as NMN repletion of NAD^+^ is important in maintaining lysosomal acidification by providing the vacuolar ATPase with ATP around lysosomal vesicles [51]. Subsequently, NMN increases ATP production in neutrophils and macrophages. However, the therapeutic effects of NMN are not associated with changes in Sectm1a expression (data not shown). Nevertheless, future research is needed to investigate how NMN enhances phagocytic and bactericidal activities of neutrophils and macrophages. Additionally, the present study revealed that NMN reduced pro-inflammatory cytokines and attenuated the infiltrations of neutrophils in major organs (heart, lung, liver and kidney) in sepsis. A recent study also reported that NMN alleviated inflammation through reprogramming macrophages in favor of their shift from the proinflammatory M1 phenotype toward anti-inflammatory M2 phenotype [21]. Thus, the suppression of bacterial dissemination and hyperinflammation may be important mechanisms by which NMN attenuates organ injury and improves survival in sepsis.

NAD^+^ is important for energy metabolism which has been implicated in the regulation of T cell function [52]. However, it is currently unknown if NAD^+^ repletion with NMN modulates T cell function in sepsis, where T cell function is suppressed in the late phase of sepsis [53], which significantly contributes to death in sepsis.

### ***NAD***^+^***repletion with NMN protects against inflammatory damage to endothelial cells***

Studies have demonstrated that microvascular dysfunction is a critical mechanism contributing to septic organ failure [54]. Endothelial cell injury including cell death, inflammation, and loss of barrier function ensures microvascular dysfunction during sepsis [55]. Studying the effects of NMN in this context, we simulated septic conditions in cardiac microvascular endothelial cells as myocardial dysfunction is very common and significantly contributes to mortality in sepsis. We found that septic conditions resulted in a reduction of intracellular NAD^+^ levels in endothelial cells, consistent with the decline of NAD^+^ levels observed in organ tissues of septic mice. The intracellular NAD^+^ levels were elevated by incubation with NMN in endothelial cells under normal and septic conditions. Septic conditions elevated mitochondrial ROS production, increased mPTP opening and caspase-3 activation, and induced barrier dysfunction in endothelial cells—all of which were prevented by NMN. Therefore, NAD^+^ repletion with NMN also protects against inflammatroy damage to endothelial cells in sepsis, in addition to limiting hyperinflammation.

### ***NAD***^+^***repletion with NMN reduces organ failure through protection of SIRT3 signaling and mitochondrial function in sepsis***

Mitochondria are sub-cellular organelles that provide energy in the form of ATP via oxidative phosphorylation (OXPHOS). Mitochondria are also associated with calcium homeostasis, intracellular ROS generation, and cell signalling functions [56]. Mitochondrial dysfunction is observed in septic patients and animal models [57, 58] and contributes to an energy crisis, oxidative stress, inflammation, and cell death, all of which contribute to organ failure in sepsis [59]. Importantly, sepsis induces a reduction of NAD^+^, which negatively affects OXPHOS as NAD^+^ is required for OXPHOS. Our previous study found that sepsis disrupted ATP synthase by calpain-mediated cleavage of its subunit ATP5A1 [60], an event facilitated by impaired SIRT3 activity due to NAD^+^ depletion [23]. Mitochondrial respiration dysfunction results in ATP depletion, which compromises the capacity of the innate immune cells (e.g. neutrophils and macrophages) to kill pathogens that cause sepsis [59]. On the other hand, a deficiency of ATP production directly induces myocardial dysfunction. Notably, the administration of NMN preserves mitochondrial NAD^+^ levels and ATP production in sepsis. Thus, the protection of mitochondrial respiration may be an important mechanism contributing to organ protection in sepsis by NAD^+^ repletion with NMN.

Mitochondrial dysfunction ensures ROS generation, which further damages the function of respiratory complexes. Mitochondrial oxidative stress observed in septic patients [61] and animal models [60] is the result of excessive ROS production due to defects in mitochondrial respiration and the impairment of antioxidant defences. In addition to the prevention of mitochondrial dysfunction, NAD^+^ repletion may also balance the redox status and promote mitochondrial antioxidant defences through SIRT1 and SIRT3 signalling. Mitochondrial oxidative stress promotes mitochondrial mPTP opening, which facilitates the release of pro-apoptotic factors and inflammatory mediators in sepsis [62], while mitochondrial ROS signals inflammatory cytokine expression. In line with this, the present study showed that NAD^+^ repletion with NMN prevented mitochondrial ROS production, mPTP opening, and pro-inflammatory response in macrophages, endothelial cells, and in vivo organs under septic conditions. In support of this finding, we previously demonstrated that scavenging mitochondrial ROS inhibits inflammatory response and apoptosis in LPS-stimulated cardiomyocytes and mouse hearts [60, 63]. Studies from others also showed that inhibition of mitochondrial ROS reduces organ dysfunction in sepsis [64–67].

An important finding of this study is that NAD^+^ repletion with NMN prevented the inhibition of SIRT3. Impaired SIRT3 activity promoted the mPTP opening, thereby contributing to mitochondrial dysfunction [24, 26]. In line with this, our in vitro study showed that NMN increased NAD^+^ levels and prevented septic conditions induced mPTP opening in endothelial cells through the SIRT3 dependent mechanism. Thus, NAD^+^ repletion with NMN may protect septic organs through SIRT3 signaling.

Although the present study was focused on mitochondria, we do not exclude the roles of non-mitochondrial NAD^+^ dependent pathways. For example, we recently reported the involvement of NAD^+^/SIRT1 in the modulation of HMGB1 during sepsis [5]. Cytosolic NAD^+^ is required for the glycolytic enzymes GAPDH and PGK1 to produce ATP around lysosomal vesicles [51], which is required for lysosomal function while normal lysosomal function is critical for cellular homeostasis and bactericidal activities of phagocytic cells. Indeed, we showed that NAD^+^ repletion with NMN improved the bactericidal activities of macrophages and neutrophils.

It is worthwhile to mention that NAD(P)^+^ and NAD(P)H systems regulate cellular metabolism and redox balance [68], both of which are compromised in sepsis. NADP^+^ is synthesized by transferring a phosphate group from ATP to NAD^+^ [68]. It has been reported that repletion of NAD^+^ is beneficial in sepsis [21, 69, 70] and that administration of thiamine elevates cellular NADP^+^ levels and provides protective effects in sepsis^71^, suggesting that thiamine and NMN may have synergetic effects. Thus, it would be plausible to examine the therapeutic effects of combining thiamine and NMN in sepsis in the future.

## Conclusion

This study demonstrates for the first time that therapeutic administration of NMN provides an anti-hyperinflammatory effect, enhances the bactericidal activity of phagocytes, and protects against inflammatory damage to endothelial cells thereby alleviating multiple organ failure and improving survival in sepsis. This study has also delineated molecular mechanisms underlying NMN’s therapeutic effects in sepsis: NMN prevents mitochondrial dysfunction and oxidative stress through SIRT3 signaling by boosting NAD^+^. Given that NMN has been used as a health supplement, and a recent clinical trial has shown support for its safety in patients, this study provides an important rationale to inform future clinical trials using NMN to treat septic organ failure.

### Supplementary Information

**Additional file 1: Table S1:** Primer sequences. **Table S2.** Organ dysfunction/injury and systemic inflammation. **Table S3.** Phagocytosis and bactericidal activities of macrophages. **Table S4.** Phagocytosis and bactericidal activities of neutrophils. **Figure S1.** L-Lactate and cytokine levels in serum. **Figure S2.** Body temperature. **Figure S3.** Histological examination of lung tissues. **Figure S4.** Flow cytometry analysis. Supplementary **Figure S5.** Cell viability in neutrophils and macrophages. **Figure S6.** TNF-α and IL-1β mRNA expression in macrophages. **Figure S7.** Cytotoxic effect of NMN in endothelial cells.

## Data Availability

Raw data are available from the corresponding author upon reasonable request.

## Abbreviations

ALT: Alanine transaminase
AST: Aspartate transaminase
BUN: Blood urea nitrogen
CCK-8: Cell counting kit-8
CFUs: Colony forming units
EBD: Evans blue dye
EF: Ejection fraction
FIP: Feces-injection-in-peritoneum
FS: Fractional shortening
LCM: LPS-conditioned medium
LPS: Lipopolysaccharides
LV: Left ventricular
LVIDd: Left ventricular end-diastolic inner diameter
LVIDs: Left ventricular end-systolic inner diameter
MCECs: Mouse cardiac microvascular endothelia cells
MDA: Myeloperoxidase
MPO: Malondialdehyde
mPTP: Mitochondrial permeability transition pore
NAD^+^: Nicotinamide adenine dinucleotide
NAMPT: Nicotinamide phosphoribosyltransferase
NMN: Nicotinamide mononucleotide
NMNAT: NMN adenylyltransferase
NR: Nicotinamide riboside
NRK1/2: NR kinase 1/2
OXPHOS: Oxidative phosphorylation
PCM: PBS-conditioned culture medium
ROS: Reactive oxygen species
SIRT3: Sirtuin 3

## References

[1] Cecconi M, Evans L, Levy M, Rhodes A (2018). Sepsis and septic shock. Lancet (London, England).

[2] Mouchiroud L, Houtkooper RH, Auwerx J (2013). NAD(+) metabolism: a therapeutic target for age-related metabolic disease. Crit Rev Biochem Mol Biol.

[3] Liaudet L, Mabley JG, Soriano FG, Pacher P, Marton A, Haskó G, Szabó C (2001). Inosine reduces systemic inflammation and improves survival in septic shock induced by cecal ligation and puncture. Am J Respir Crit Care Med.

[4] Kwon WY, Suh GJ, Kim KS, Kwak YH (2011). Niacin attenuates lung inflammation and improves survival during sepsis by downregulating the nuclear factor-kappaB pathway. Crit Care Med.

[5] Hong G, Zheng D, Zhang L, Ni R, Wang G, Fan GC, Lu Z, Peng T (2018). Administration of nicotinamide riboside prevents oxidative stress and organ injury in sepsis. Free Radical Biol Med.

[6] Belenky P, Bogan KL, Brenner C (2007). NAD+ metabolism in health and disease. Trends Biochem Sci.

[7] Yagi M, Toshima T, Amamoto R, Do Y, Hirai H, Setoyama D, Kang D, Uchiumi T (2021). Mitochondrial translation deficiency impairs NAD(+) -mediated lysosomal acidification. EMBO J.

[8] Klimova N, Fearnow A, Long A, Kristian T (2020). NAD(+) precursor modulates post-ischemic mitochondrial fragmentation and reactive oxygen species generation via SIRT3 dependent mechanisms. Exp Neurol.

[9] Liu S, She F, Zhang W, Hu X, Zhao X, Yao Y (2020). Tryptophan decreases the intensity of lipopolysaccharide-induced acute lung injury in a rat model. Amino Acids.

[10] Zeden JP, Fusch G, Holtfreter B, Schefold JC, Reinke P, Domanska G, Haas JP, Gruendling M, Westerholt A, Schuett C (2010). Excessive tryptophan catabolism along the kynurenine pathway precedes ongoing sepsis in critically ill patients. Anaesth Intensive Care.

[11] Yuan H, Wan J, Li L, Ge P, Li H, Zhang L (2012). Therapeutic benefits of the group B3 vitamin nicotinamide in mice with lethal endotoxemia and polymicrobial sepsis. Pharmacol Res.

[12] Bitterman KJ, Anderson RM, Cohen HY, Latorre-Esteves M, Sinclair DA (2002). Inhibition of silencing and accelerated aging by nicotinamide, a putative negative regulator of yeast sir2 and human SIRT1. J Biol Chem.

[13] Bogan KL, Brenner C (2008). Nicotinic acid, nicotinamide, and nicotinamide riboside: a molecular evaluation of NAD+ precursor vitamins in human nutrition. Annu Rev Nutr.

[14] Dollerup OL, Christensen B, Svart M, Schmidt MS, Sulek K, Ringgaard S, Stodkilde-Jorgensen H, Moller N, Brenner C, Treebak JT, Jessen N (2018). A randomized placebo-controlled clinical trial of nicotinamide riboside in obese men: safety, insulin-sensitivity, and lipid-mobilizing effects. Am J Clin Nutr.

[15] Irie J, Inagaki E, Fujita M, Nakaya H, Mitsuishi M, Yamaguchi S, Yamashita K, Shigaki S, Ono T, Yukioka H, Okano H, Nabeshima YI, Imai SI, Yasui M, Tsubota K, Itoh H (2020). Effect of oral administration of nicotinamide mononucleotide on clinical parameters and nicotinamide metabolite levels in healthy Japanese men. Endocr J.

[16] Belenky P, Racette FG, Bogan KL, McClure JM, Smith JS, Brenner C (2007). Nicotinamide riboside promotes Sir2 silencing and extends lifespan via Nrk and Urh1/Pnp1/Meu1 pathways to NAD+. Cell.

[17] Hong W, Mo F, Zhang Z, Huang M, Wei X (2020). Nicotinamide mononucleotide: a promising molecule for therapy of diverse diseases by targeting NAD+ metabolism. Front Cell Dev Biol.

[18] Liu J, Zong Z, Zhang W, Chen Y, Wang X, Shen J, Yang C, Liu X, Deng H (2021). Nicotinamide mononucleotide alleviates LPS-induced inflammation and oxidative stress via decreasing COX-2 expression in macrophages. Front Mol Biosci.

[19] Tian Y, Zhu CL, Li P, Li HR, Liu Q, Deng XM, Wang JF (2022). Nicotinamide mononucleotide attenuates LPS-induced acute lung injury with anti-inflammatory, anti-oxidative and anti-apoptotic effects. J Surg Res.

[20] He S, Gao Q, Wu X, Shi J, Zhang Y, Yang J, Li X, Du S, Yu J (2022). NAD(+) ameliorates endotoxin-induced acute kidney injury in a sirtuin1-dependent manner via GSK-3beta/Nrf2 signalling pathway. J Cell Mol Med.

[21] Cros C, Margier M, Cannelle H, Charmetant J, Hulo N, Laganier L, Grozio A, Canault M (2022). Nicotinamide mononucleotide administration triggers macrophages reprogramming and alleviates inflammation during sepsis induced by experimental peritonitis. Front Mol Biosci.

[22] Bernardi P, Rasola A, Forte M, Lippe G (2015). The mitochondrial permeability transition pore: channel formation by F-ATP synthase, integration in signal transduction, and role in pathophysiology. Physiol Rev.

[23] Koentges C, Cimolai MC, Pfeil K, Wolf D, Marchini T, Tarkhnishvili A, Hoffmann MM, Odening KE, Diehl P, von Zur MC, Alvarez S, Bode C, Zirlik A, Bugger H (2019). Impaired SIRT3 activity mediates cardiac dysfunction in endotoxemia by calpain-dependent disruption of ATP synthesis. J Mol Cell Cardiol.

[24] Fonai F, Priber JK, Jakus PB, Kalman N, Antus C, Pollak E, Karsai G, Tretter L, Sumegi B, Veres B (2015). Lack of cyclophilin D protects against the development of acute lung injury in endotoxemia. Biochem Biophys Acta.

[25] Sun F, Si Y, Bao H, Xu Y, Pan X, Zeng L, Jing L (2017). Regulation of Sirtuin 3-mediated deacetylation of cyclophilin D attenuated cognitive dysfunction induced by sepsis-associated encephalopathy in mice. Cell Mol Neurobiol.

[26] Veres B, Eros K, Antus C, Kalman N, Fonai F, Jakus PB, Boros E, Hegedus Z, Nagy I, Tretter L, Gallyas F, Sumegi B (2021). Cyclophilin D-dependent mitochondrial permeability transition amplifies inflammatory reprogramming in endotoxemia. FEBS Open Bio.

[27] Kobayashi T, Uchino H, Elmer E, Ogihara Y, Fujita H, Sekine S, Ishida Y, Saiki I, Shibata S, Kawachi A (2022). Disease outcome and brain metabolomics of cyclophilin-D knockout mice in sepsis. Int J Mol Sci.

[28] Zheng D, Yu Y, Li M, Wang G, Chen R, Fan GC, Martin C, Xiong S, Peng T (2016). Inhibition of microRNA 195 prevents apoptosis and multiple-organ injury in mouse models of sepsis. J Infect Dis.

[29] Zheng D, Cao T, Zhang LL, Fan GC, Qiu J, Peng TQ (2021). Targeted inhibition of calpain in mitochondria alleviates oxidative stress-induced myocardial injury. Acta Pharmacol Sin.

[30] Green TP, Johnson DE, Marchessault RP, Gatto CW (1988). Transvascular flux and tissue accrual of Evans blue: effects of endotoxin and histamine. J Lab Clin Med.

[31] Rui T, Feng Q, Lei M, Peng T, Zhang J, Xu M, Abel ED, Xenocostas A, Kvietys PR (2005). Erythropoietin prevents the acute myocardial inflammatory response induced by ischemia/reperfusion via induction of AP-1. Cardiovasc Res.

[32] Vong L, Sherman PM, Glogauer M (2013). Quantification and visualization of neutrophil extracellular traps (NETs) from murine bone marrow-derived neutrophils. Methods Mol Biol.

[33] Graesser D, Solowiej A, Bruckner M, Osterweil E, Juedes A, Davis S, Ruddle NH, Engelhardt B, Madri JA (2002). Altered vascular permeability and early onset of experimental autoimmune encephalomyelitis in PECAM-1-deficient mice. J Clin Invest.

[34] Cao T, Fan S, Zheng D, Wang G, Yu Y, Chen R, Song LS, Fan GC, Zhang Z, Peng T (2019). Increased calpain-1 in mitochondria induces dilated heart failure in mice: role of mitochondrial superoxide anion. Basic Res Cardiol.

[35] Mu X, Wang P, Wang X, Li Y, Zhao H, Li Q, Essandoh K, Deng S, Peng T, Fan GC (2020). Identification of a novel antisepsis pathway: Sectm1a enhances macrophage phagocytosis of bacteria through activating GITR. J Immunol.

[36] Fine N, Barzilay O, Glogauer M (2017). Analysis of human and mouse neutrophil phagocytosis by flow cytometry. Methods Mol Biol.

[37] Novakowski KE, Loukov D, Chawla V, Bowdish DM (2017). Bacterial Binding, Phagocytosis, and Killing: Measurements Using Colony Forming Units. Methods Mol Biol.

[38] Yoshino J, Baur JA, Imai SI (2018). NAD(+) intermediates: the biology and therapeutic potential of NMN and NR. Cell Metab.

[39] Grozio A, Mills KF, Yoshino J, Bruzzone S, Sociali G, Tokizane K, Lei HC, Cunningham R, Sasaki Y, Migaud ME, Imai SI (2019). Slc12a8 is a nicotinamide mononucleotide transporter. Nat Metab.

[40] Nadeeshani H, Li J, Ying T, Zhang B, Lu J (2022). Nicotinamide mononucleotide (NMN) as an anti-aging health product—Promises and safety concerns. J Adv Res.

[41] Nair AB, Jacob S (2016). A simple practice guide for dose conversion between animals and human. J Basic Clin Pharm.

[42] Ince C, Mayeux PR, Nguyen T, Gomez H, Kellum JA, Ospina-Tascón GA, Hernandez G, Murray P, De Backer D (2016). The endothelium in sepsis. Shock.

[43] Andrews T, Sullivan KE (2003). Infections in patients with inherited defects in phagocytic function. Clin Microbiol Rev.

[44] Danikas DD, Karakantza M, Theodorou GL, Sakellaropoulos GC, Gogos CA (2008). Prognostic value of phagocytic activity of neutrophils and monocytes in sepsis. Correlation to CD64 and CD14 antigen expression. Clin Exp Immunol..

[45] Kovach MA, Standiford TJ (2012). The function of neutrophils in sepsis. Curr Opin Infect Dis.

[46] Shen XF, Cao K, Jiang JP, Guan WX, Du JF (2017). Neutrophil dysregulation during sepsis: an overview and update. J Cell Mol Med.

[47] Patel JM, Sapey E, Parekh D, Scott A, Dosanjh D, Gao F, Thickett DR (2018). Sepsis induces a dysregulated neutrophil phenotype that is associated with increased mortality. Mediators Inflamm.

[48] Hotchkiss RS, Monneret G, Payen D (2013). Immunosuppression in sepsis: a novel understanding of the disorder and a new therapeutic approach. Lancet Infect Dis.

[49] Mu X, Fan H, Wang P, Li Y, Domenico K, Li Q, Wang X, Essandoh K, Chen J, Peng T, Fan GC (2021). Sectm1a facilitates protection against inflammation-induced organ damage through promoting TRM self-renewal. Mol Ther.

[50] Li Y, Deng S, Wang X, Huang W, Chen J, Robbins N, Mu X, Essandoh K, Peng T, Jegga AG, Rubinstein J, Adams DE, Wang Y, Peng J, Fan GC (2021). Sectm1a deficiency aggravates inflammation-triggered cardiac dysfunction through disruption of LXRalpha signalling in macrophages. Cardiovasc Res.

[51] Yagi M, Toshima T, Amamoto R, Do Y, Hirai H, Setoyama D, Kang D, Uchiumi T (2021). Mitochondrial translation deficiency impairs NAD mediated lysosomal acidification. EMBO J.

[52] Wang Y, Wang F, Wang L, Qiu S, Yao Y, Yan C, Xiong X, Chen X, Ji Q, Cao J, Gao G, Li D, Zhang L, Guo Z, Wang R, Wang H, Fan G (2021). NAD(+) supplement potentiates tumor-killing function by rescuing defective TUB-mediated NAMPT transcription in tumor-infiltrated T cells. Cell Rep.

[53] Jensen IJ, Sjaastad FV, Griffith TS, Badovinac VP (2018). Sepsis-Induced T cell immunoparalysis: the ins and outs of impaired T cell immunity. J Immunol.

[54] Vincent JL, De Backer D (2005). Microvascular dysfunction as a cause of organ dysfunction in severe sepsis. Crit Care.

[55] Joffre J, Hellman J, Ince C, Ait-Oufella H (2020). Endothelial Responses in Sepsis. Am J Respir Crit Care Med.

[56] van der Bliek AM, Sedensky MM, Morgan PG (2017). Cell biology of the mitochondrion. Genetics.

[57] Brealey D, Brand M, Hargreaves I, Heales S, Land J, Smolenski R, Davies NA, Cooper CE, Singer M (2002). Association between mitochondrial dysfunction and severity and outcome of septic shock. Lancet.

[58] Brealey D, Karyampudi S, Jacques TS, Novelli M, Stidwill R, Taylor V, Smolenski RT, Singer M (2004). Mitochondrial dysfunction in a long-term rodent model of sepsis and organ failure. Am J Physiol Regul Integr Comp Physiol.

[59] Singer M (2014). The role of mitochondrial dysfunction in sepsis-induced multi-organ failure. Virulence.

[60] Ni R, Zheng D, Wang Q, Yu Y, Chen R, Sun T, Wang W, Fan GC, Greer PA, Gardiner RB, Peng T (2015). Deletion of capn4 protects the heart against endotoxemic injury by preventing ATP synthase disruption and inhibiting mitochondrial superoxide generation. Circ Heart Fail.

[61] Ayala JC, Grismaldo A, Aristizabal-Pachon AF, Mikhaylenko EV, Nikolenko VN, Mikhaleva LM, Somasundaram SG, Kirkland CE, Aliev G, Morales L (2021). Mitochondrial dysfunction in intensive care unit patients. Curr Pharm Des.

[62] Kozlov AV, Bahrami S, Calzia E, Dungel P, Gille L, Kuznetsov AV, Troppmair J (2011). Mitochondrial dysfunction and biogenesis: do ICU patients die from mitochondrial failure?. Ann Intensive Care.

[63] Zhu H, Shan L, Schiller PW, Mai A, Peng T (2010). Histone deacetylase-3 activation promotes tumor necrosis factor-alpha (TNF-alpha) expression in cardiomyocytes during lipopolysaccharide stimulation. J Biol Chem.

[64] Patil NK, Parajuli N, MacMillan-Crow LA, Mayeux PR (2014). Inactivation of renal mitochondrial respiratory complexes and manganese superoxide dismutase during sepsis: mitochondria-targeted antioxidant mitigates injury. Am J Physiol Renal Physiol.

[65] Ramsey H, Wu MX (2014). Mitochondrial anti-oxidant protects IEX-1 deficient mice from organ damage during endotoxemia. Int Immunopharmacol.

[66] Wu J, Zhang M, Hao S, Jia M, Ji M, Qiu L, Sun X, Yang J, Li K (2015). Mitochondria-targeted peptide reverses mitochondrial dysfunction and cognitive deficits in sepsis-associated encephalopathy. Mol Neurobiol.

[67] Yao X, Carlson D, Sun Y, Ma L, Wolf SE, Minei JP, Zang QS (2015). Mitochondrial ROS Induces cardiac inflammation via a pathway through mtDNA damage in a pneumonia-related sepsis model. PLoS ONE.

[68] Xiao W, Wang RS, Handy DE, Loscalzo J (2018). NAD(H) and NADP(H) redox couples and cellular energy metabolism. Antioxid Redox Signal.

[69] Li HR, Liu Q, Zhu CL, Sun XY, Sun CY, Yu CM, Li P, Deng XM, Wang JF (2023). β-Nicotinamide mononucleotide activates NAD+/SIRT1 pathway and attenuates inflammatory and oxidative responses in the hippocampus regions of septic mice. Redox Biol.

[70] He S, Gao Q, Wu X, Shi J, Zhang Y, Yang J, Li X, Du S, Zhang Y, Yu J (2022). NAD(+) ameliorates endotoxin-induced acute kidney injury in a sirtuin1-dependent manner via GSK-3β/Nrf2 signalling pathway. J Cell Mol Med.

[71] Costa NA, Pereira AG, Sugizaki CSA, Vieira NM, Garcia LR, de Paiva SAR, Zornoff LAM, Azevedo PS, Polegato BF, Minicucci MF (2021). Insights Into thiamine supplementation in patients with septic shock. Front Med.

