# Supplementary material for: Nicotinamide mononucleotide as a therapeutic agent to alleviate multi-organ failure in sepsis
Source: J Transl Med. 2023 Dec 6;21:883. doi: 10.1186/s12967-023-04767-3 (PMC10699070; doi:10.1186/s12967-023-04767-3)
Supplement: Supplementary file 1 — Additional file 1: Table S1: Primer sequences. Table S2. Organ dysfunction/injury and systemic inflammation. Table S3. Phagocytosis and bactericidal activities of macrophages. Table S4. Phagocytosis and bactericidal activities of neutrophils. Figure S1. L-Lactate and cytokine levels in serum. Figure S2. Body temperature. Figure S3. Histological examination of lung tissues. Figure S4. Flow cytometry analysis. Supplementary Figure S5. Cell viability in neutrophils and macrophages. Figure S6. TNF-α and IL-1β mRNA expression in macrophages. Figure S7. Cytotoxic effect of NMN in endothelial cells. [file 12967_2023_4767_MOESM1_ESM.docx]

**Supplementary Materials**

1. Supplementary Table 1
2. Supplementary Table 2
3. Supplementary Table 3
4. Supplementary Table 4
5. Supplementary Figures 1-7 and Figure Legends

s

Supplementary Table 1. Primer sequences

| **Gene** |  |  | **Oligonucleotide sequence (5’-3’)** |
| --- | --- | --- | --- |
| NRK1 |  |  | Forward: AGGGAAGACGACACTGGCTA |
|  |  |  | Reverse: CTGGGTTTTCCATCCAACAG |
| NRK2 |  |  | Forward: CCAAATAGCAGTCGGAGAGG |
|  |  |  | Reverse: GCTGTACAAGTCCACCAGGG |
| Slc12a8 |  |  | Forward: CACAGGGGCCATGATGACTT |
|  |  |  | Reverse: CTTGAAGCAGGGCTCTGTCA |
| TNF-α |  |  | Forward: CGGTGCCTATGTCTCAGCCT |
|  |  |  | Reverse: GAGGGTCTGGGCCATAGAAC |
| IL-1β |  |  | Forward: TCACAGCAGCACATCAACAA |
|  |  |  | Reverse: TGTCCTCATCCTGGAAGGT |
| iNOS |  |  | Forward: GATGGTCCGCAAGAGAGTGC |
|  |  |  | Reverse: AACGTA GACCTTGGGTTTGCC |
| VCAM1 |  |  | Forward: TTAAAGTCTGTGGATGGCTCGTAC |
|  |  |  | Reverse: CTTAATTGTCAGCCAACTTCAGTCTT |
| GAPDH |  |  | Forward: AAAGGGCATCCTGGGCTACA |
|  |  |  | Reverse: CAGTGTTGGGGGCTGAGTTG |

**Supplementary Figures**

**Supplementary Figure 1. L-Lactate and cytokine levels in serum.** A single dose of NMN (500 mg/kg, i.p.) was administrated into mice after an hour of FIP. Six hours after FIP, serum was collected. The levels of L-Lactate (A), TNF-α (B), IL-1β (C), IL-2 (D) and IL-10 (E) were determined. (F) The levels of L-Lactate in serum were determined at 24 hours after FIP. Data are mean ± SD, n = 8 in each group. **P*＜0.05 vs saline + vehicle and #*P*＜0.05 vs feces + vehicle (One-way analysis of variance followed by Newman–Keuls test).

**Supplementary Figure 2. Body temperature.**  A single dose of NMN (500 mg/kg, i.p.) was administrated into mice after an hour of FIP. The body surface temperature was monitored at 6, 12, 18 and 24 hours after FIP. Data are mean ± SD, n= 8 mice in each group.

**Supplementary Figure3.** Histological examination of lung tissues. Mice received saline or feces (0.8g feces/kg body weight, i.p.). 24 hours later, lung tissues were fixed, embedded and sectioned. H&E staining was performed. Representative microphotographs for patho-histological changes in lung from sham and FIP-induced septic mice. Seven mice were included in each group.

**Supplementary Figure 4. Flow cytometry analysis.**  A single dose of NMN (500 mg/kg, i.p.) was administrated into mice after an hour of FIP. In vivo neutrophil phagocytosis in peritoneum was analyzed at 6 hours and 24 hours after FIP, respectively. Representative flow cytometry dot plots of neutrophils from vehicle and NMN-treated mouse peritoneum are presented at 6 hours (A) and 24 hours after FIP (B).

**Supplementary Figure 5. Cell viability in neutrophils and macrophages.** Neutrophils and macrophages were pretreated with NMN and then living *E. coli* or saline. Cell viability of neutrophils (A) and macrophages (B) were analyzed by CCK-8 assay. Data are mean ± SD from 4 different cultures. **P*＜0.05 vs saline + vehicle (One-way analysis of variance followed by Newman–Keuls test).

**Supplementary Figure 6. TNF-α and IL-1β mRNA expression in macrophages.** Primary peritoneal macrophages were incubated with LPS (100 ng/mL) or saline in the presence of NMN (500 µM) or vehicle for 24 hours. The levels of TNF-α and IL-1β mRNA were determined and normalized to GAPDH. Data are mean ± SD, n = 5 independent isolations in each group. **P*＜0.05 vs saline + vehicle and #*P*＜0.05 vs LPS + vehicle (One-way analysis of variance followed by Newman–Keuls test).

**Supplementary Figure 7. Cytotoxic effect of NMN in endothelial cells.** Mouse cardiac microvascular endothelial cells (MCECs) were incubated with NMN at different concentrations (0, 0.5, 1. 2, and 5 mM) for 24 hours. The LDH release was analyzed in culture medium. Data are mean ± SD, n = 4 independent cell cultures. **P*＜0.05 vs 0 (One-way analysis of variance followed by Newman–Keuls test).
